# Diagnostic performance of metagenomic next-generation sequencing for Pneumocystis jirovecii pneumonia

**DOI:** 10.1186/s12879-023-08440-4

**Published:** 2023-07-10

**Authors:** Xuefang Li, Zhijun Li, Jian Ye, Wu Ye

**Affiliations:** 1 Department of Infectious Diseases, Zhejiang Hospital, 1229 Gudun Road, Xihu District, Hangzhou, 310013 Zhejiang Province People’s Republic of China; 2 Department of Respiratory Diseases, Zhejiang Hospital, 1229 Gudun Road, Xihu District, Hangzhou, 310013 Zhejiang Province People’s Republic of China

**Keywords:** Pneumocystis jirovecii pneumonia, Metagenomic next-generation sequencing, Diagnosis, meta-analysis, Bronchoalveolar lavage fluid

## Abstract

**Objective:**

Pneumocystis jirovecii pneumonia (PJP) can be a life-threatening opportunistic infection. We aimed to evaluate the diagnostic accuracy of metagenomic next-generation sequencing (mNGS) for PJP.

**Methods:**

A comprehensive electronic literature search of Web of Knowledge, PubMed, Cochrane Library, CNKI and Wanfang data was performed. Bivariate analysis was conducted to calculate the pooled sensitivity, specificity, diagnostic odds ratio (DOR), the area under the summary receiver operator characteristic (SROC) curve and the Q-point value (Q*).

**Results:**

The literature search resulted in 9 studies with a total of 1343 patients, including 418 cases diagnosed with PJP and 925 controls. The pooled sensitivity of mNGS for diagnosis of PJP was 0.974 [95% confidence interval (CI), 0.953–0.987]. The pooled specificity was 0.943 (95% CI, 0.926–0.957), the DOR was 431.58 (95% CI, 186.77-997.27), the area under the SROC curve was 0.987, and the Q* was 0.951. The *I*^2^ test indicated no heterogeneity between studies. The Deek funnel test suggested no potential publication bias. Subgroup analyses showed that the area under the SROC curve of mNGS for diagnosis of PJP in immunocompromised and non-HIV patients was 0.9852 and 0.979, respectively.

**Conclusions:**

Current evidence indicates that mNGS exhibits excellent accuracy for the diagnosis of PJP. The mNGS is a promising tool for assessment of PJP in both immunocompromised and non-HIV patients.

## Introduction

Pneumocystis jirovecii is an opportunistic fungal pathogen that may cause life-threatening pneumonia in immunodeficient hosts [1, 2]. Pneumocystis jirovecii pneumonia (PJP) was first found in immunocompromised children during the Second World War and later widely recognized in adults infected with human immunodeficiency virus (HIV) during the epidemic of acquired immunodeficiency syndrome (AIDS) [3]. Although the incidence of PJP in patients with HIV infection decreased after the clinical usage of antiretroviral therapy, PJP remains one of the most severe and common infections in patients with AIDS [4, 5]. In recent years, the morbidity rate of PJP has been increasing in non-HIV individuals with autoimmune diseases, solid organ or stem cell transplants, and hematologic malignancies [6, 7]. Immunocompromised patients without HIV are characterized by faster disease progression, with a mortality rate of 35–55% compared with 10–20% in HIV cases [8].

Patients with PJP typically present with fever, dry cough, rapidly progressive dyspnea and respiratory failure. However, clinical features of PJP are nonspecific [9]. Traditionally, the diagnosis of PJP relies on comprehensive analyses of clinical manifestations, imaging findings, and microbiologic tests of sputum or bronchoalveolar lavage fluid (BALF) for Pneumocystis jirovecii [3, 10]. In non-HIV immunocompromised patients, the sensitivity of conventional microbiological tests for sputum specimens is low, ranging from 38 to 53% [11]. Positive results of BALF and lung biopsy samples are considered as the “gold standard” for the diagnosis of PJP [12].

In vitro culture of Pneumocystis jirovecii is extremely difficult. Microbial diagnosis of PJP is usually based on direct-view techniques with different staining tests or immunofluorescence methods [13]. However, traditional diagnostic tests have proven to be insensitive and may rely on invasive procedures to obtain adequate samples.To address these issues, molecular tests such as antibody-antigen assays, loop-mediated isothermal amplification (LAMP) and polymerase chain reaction (PCR) have been developed. These techniques are sensitive and may be able to test life forms of Pneumocystis jirovecii in non-invasive specimens such as serum,oral rinses, sputum, and nasopharyngeal aspirates [3, 14]. PJP is a severe condition associated with a high mortality, ranging from 27 to 55% [13]. Early diagnosis of PJP is critical for improving clinical outcomes [3].

In recent years, with the advancement of molecular diagnostic techniques, metagenomic next-generation sequencing (mNGS) technology has been developed to provide information on the DNA sequence of microbial genomes [15]. The most attractive and important advantage of mNGS is its ability to pick up all pathogens, including viruses, bacteria, fungi, and parasites [16]. Among cases of fungal infections diagnosed by mNGS, Pneumocystis jirovecii is the main pathogen, accounting for approximately 25% [16]. Recent studies showed that mNGS might be a excellent tool for diagnosis of PJP [17–25]. Therefore, we performed a meta-analysis of eligible studies to assess diagnostic accuracy of mNGS for PJP, and conducted subgroup analyses to investigate the performance of mNGS in immunocompromised and non-HIV patients.

## Methods

### Literature search strategy

We searched Web of Knowledge, PubMed, Cochrane Library, CNKI, and Wanfang data to identify studies evaluating diagnostic efficacy of mNGS for PJP published up to November 2022. Reference lists of relevant reviews and included studies were manually retrieved. The following search terms were employed: “pneumocystis”, “pneumonia, pneumocystis”, “PJP”, “PCP”, “high-throughput nucleotide sequencing”, “next generation sequencing”, “metagenomic next-generation sequencing”, and “mNGS”. No ethics approval is required as all our analyses are based on previously published data.

### Research selection

We included studies that met the following criteria: (1) studies restricted to human subjects; (2) original research published in English or Chinese; (3) papers evaluated diagnostic efficacy of mNGS for PJP; (4) trials enrolled at least 10 patients with PJP; (5) studies provided sufficient data to calculate the number of true positive (TP), false negative (FN), false positive (FP), and true negative (TN).

### Data extraction and quality assessment

Two reviewers (Xuefang Li and Wu Ye) independently reviewed eligible studies and extracted relevant data. In case of disagreement, two authors reassessed discrepancies and resolved them by consensus. The following data were obtained: surname of the first author, year of publication, age and size of the study population, research type, sample source, sequence methods, sequence platforms, diagnostic criteria, immunocompromised conditions, HIV infection, and number of TP, FP, FN and TN.

Quality assessment is important in meta-analyses of diagnostic accuracy studies [26]. Two authors (Xuefang Li and Wu Ye) independently performed quality assessment of the included studies using the Quality Assessment of Diagnostic Accuracy Studies(QUADAS) [27]. We assigned a score of 0 point for each “no”, 0.5 for each “unclear”, and 1 for each “yes”. The highest QUADAS score is 14 points [27].

### Statistical analysis

We conducted all statistical analyses using the Stata 16.0 software (StataCorp, College Station, TX) and MetaDisc 1.4 software (Clinical Biostatistics Team, Ramón y Cajal Hospital in Madrid, Spain). Bivariate analysis was performed to calculate the pooled sensitivity, specificity, positive likelihood ratio (PLR), negative likelihood ratio (NLR), and diagnostic odds ratio (DOR). Higher values of DOR suggest better performance of diagnostic tests [28]. We constructed the summary receiver operator characteristic (SROC) curve to calculate the area under the SROC curve and the Q-point value (Q*). The area under the SROC curve indicates overall accuracy of screening tests [28]. The Q* point on the SROC curve represents the maximum joint specificity and sensitivity [28]. The Cochrane-*Q* test and the inconsistency index *I*^2^ statistic were used to assess the heterogeneity among included trials. *I*^2^ value > 50% is suggestive of substantial heterogeneity. Publication bias was estimated by the Deek funnel plot [29]. Statistical significance was set at *P* < 0.05.

## Results

### Study characteristics

**Fig. 1:**
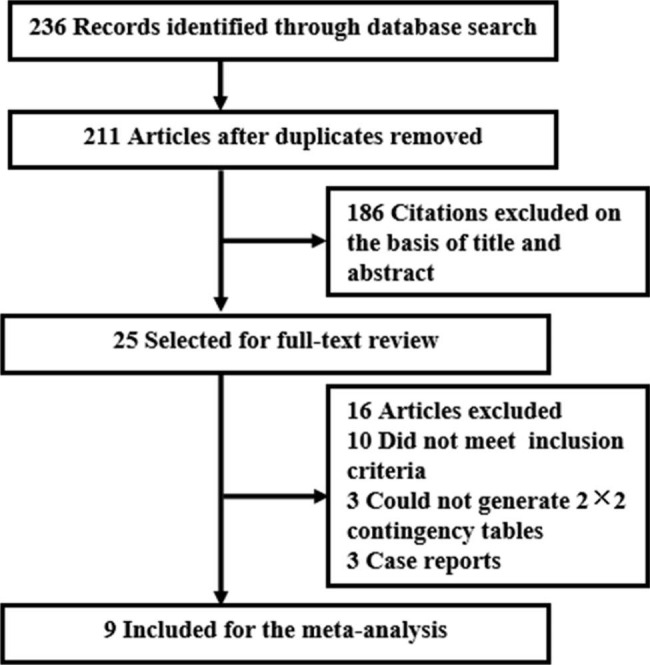
Flow diagram of the study selection process

The initial search yielded 236 citations. One hundred eighty-six references were excluded on basis of title and abstract. Twenty five articles were selected for full-text review. Ultimately, our meta-analysis enrolled 9 studies [17–25] with a total of 1343 patients, including 418 cases diagnosed with PJP and 925 controls (Fig. 1). All participants were adults. As shown in Table 1, retrospective design was performed in all trials. Five studies [17, 18, 20–22] only detected BALF samples, 4 merely included immunocompromised patients [20, 21, 24, 25], and 3 only enrolled non-HIV cases [19, 21, 23]. In our meta-analysis, the included studies all had QUADAS scores > 10, suggesting that eligible trials were of high quality.

**Table 1:** Characteristics of eligible studies in the present meta-analysis

Study	Age of patients	Patients (n)	Research type	Sample source	Sequence methods	Sequence platforms	Diagnostic criteria	All patients were immunosuppressed	Non-HIV infection	TP	FP	FN	TN	QUADAS
Wu et al. , 2022 [17]	Adult	502	Retrospective	BALF	DNA-Seq	MGISEQ	CM, CR, CMT, mNGS, TR	No	No	35	17	0	450	12
Shi et al. , 2022 [18]	Adult	58	Retrospective	BALF	DNA-Seq/RNA-Seq	BGISEQ	CM, CR, LT, CMT, mNGS, TR	No	Unknown	22	0	2	34	12
Wang et al. , 2022 [19]	Adult	189	Retrospective	BALF/ Blood	DNA-Seq	MGISEQ/ NextSeq	IC, CM, CR, CMT, mNGS	No	Yes	122	6	0	61	13
Lin et al. , 2022 [20]	Adult	69	Retrospective	BALF	DNA-Seq	MGISEQ	CM, LT, CR, CMT, mNGS, TR	Yes	Unknown	25	6	0	38	11
Sun et al. , 2022 [21]	Adult	198	Retrospective	BALF	DNA-Seq	Nextseq	IC, CM, CR, LT, CMT	Yes	Yes	75	18	2	103	13
Liu et al. , 2021 [22]	Adult	47	Retrospective	BALF	DNA-Seq	Unknown	CM, CR, mNGS	No	No	20	1	4	22	12
Jiang et al. , 2021 [23]	Adult	194	Retrospective	BALF/ Blood	DNA-Seq	MGISEQ	IC, CM, CR, CMT, mNGS	No	Yes	60	5	0	129	12
Zhang et al. , 2021 [24]	Adult	24	Retrospective	BALF/Sputum/Blood	DNA-Seq	NextSeq	CMT, mNGS	Yes	Unknown	13	0	1	10	12
Gu et al. , 2020 [25]	Adult	62	Retrospective	Blood	Unknown	BGISEQ	CM, CR, LT	Yes	Unknown	35	0	2	25	12

BALF, bronchoalveolar lavage fluid; DNA-Seq, DNA sequence; HIV, human immunodeficiency virus; TP, true positive, FN, false negative; FP, false positive; TN, true negative; QUADAS, Quality Assessment of Diagnostic Accuracy Studies; IC, immunocompromised conditions; CM, clinical manifestations; LT, laboratory tests; .CR, chest radiology; CMT, conventional microbiological tests; mNGS, metagenomic next-generation sequencing; TR, treatment response

### Diagnostic accuracy of metagenomic next-generation sequencing for Pneumocystis jirovecii pneumonia

**Fig. 2:**
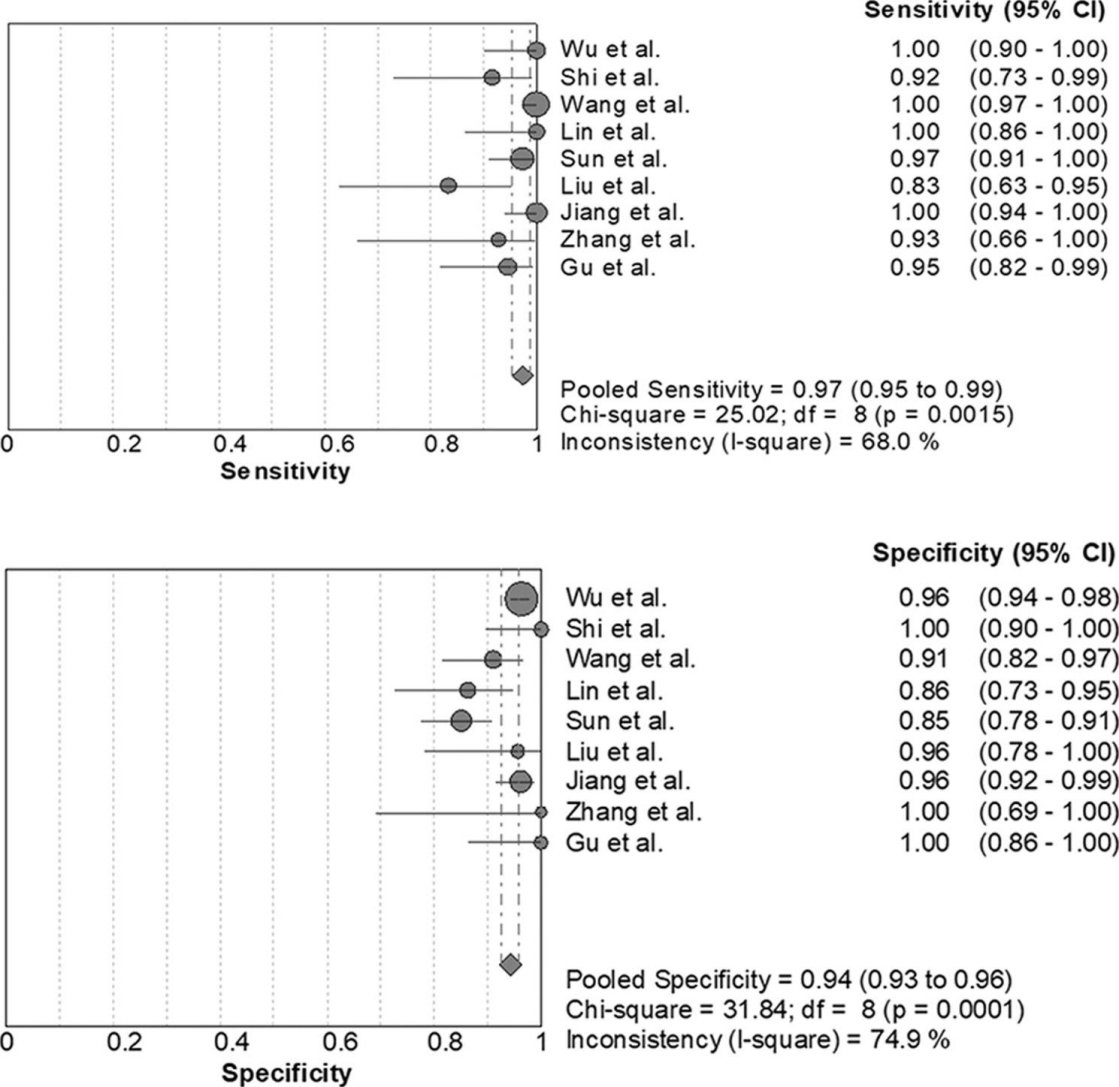
Forest plot of sensitivity and specificity for metagenomic next-generation sequencing in diagnosis of Pneumocystis jirovecii pneumonia. The pooled sensitivity was 0.974 (95% CI, 0.953–0.987) and the pooled specificity was 0.943 (95% CI, 0.926–0.957)

**Fig. 3:**
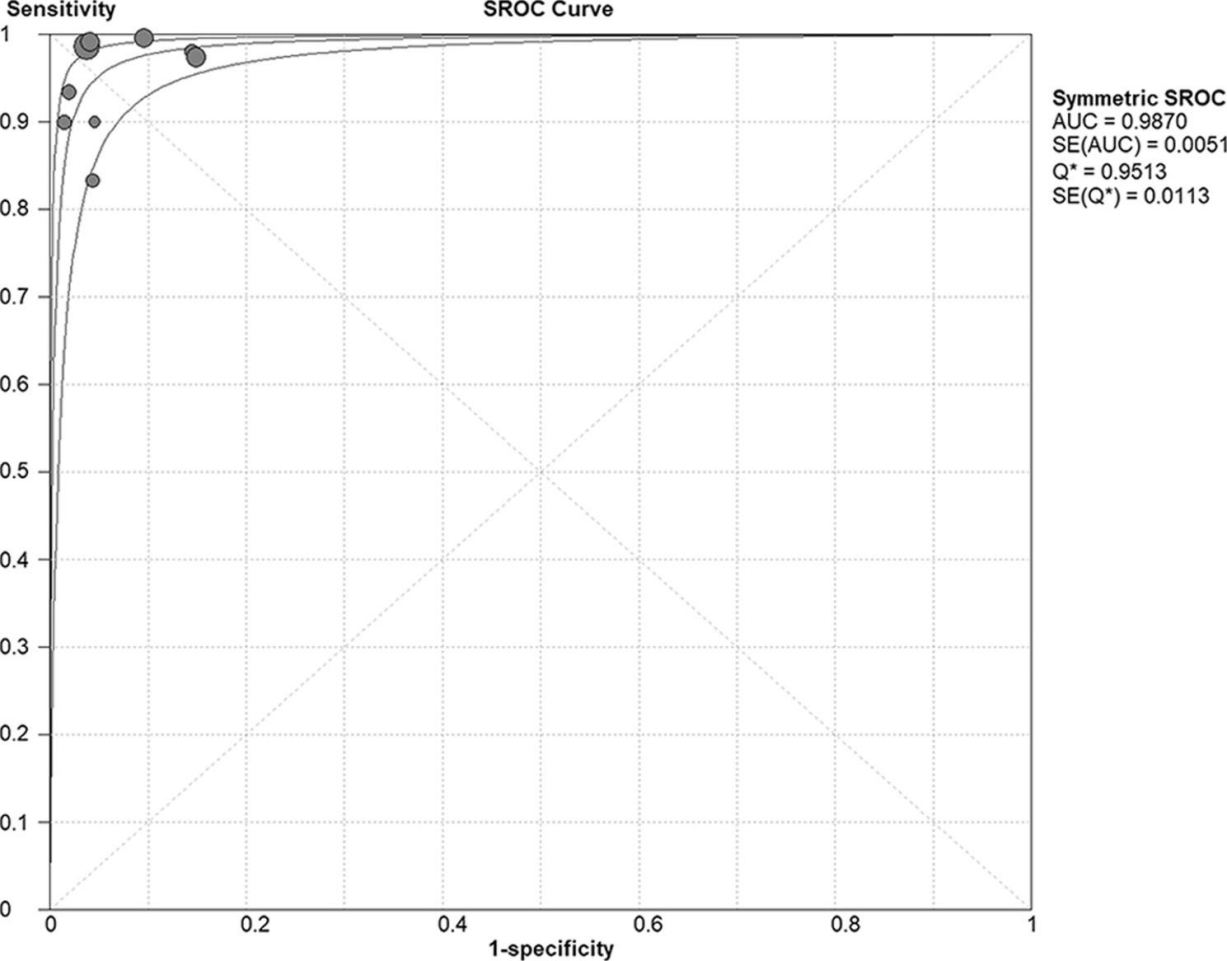
Summary receiver operating characteristic (SROC) curve of metagenomic next-generation sequencing in diagnosis of Pneumocystis jirovecii pneumonia. The area under the SROC curve was 0.987, and the Q* was 0.951

The forest plot presented in Fig. 2 showed that the pooled sensitivity of mNGS for diagnosis of PJP was 0.974 [95% confidence interval (CI), 0.953–0.987]. The pooled specificity was 0.943 (95% CI, 0.926–0.957), the PLR was 14.344 (95% CI, 8.127–25.317), and the NLR was 0.048 (95% CI, 0.019–0.117).The pooled DOR was 431.58 (95% CI, 186.77-997.27), the area under the SROC curve was 0.987, and the Q* was 0.951 (Fig. 3). These results indicated that mNGS had excellent value for diagnosis of PJP.

### Heterogeneity and publication bias

**Fig. 4:**
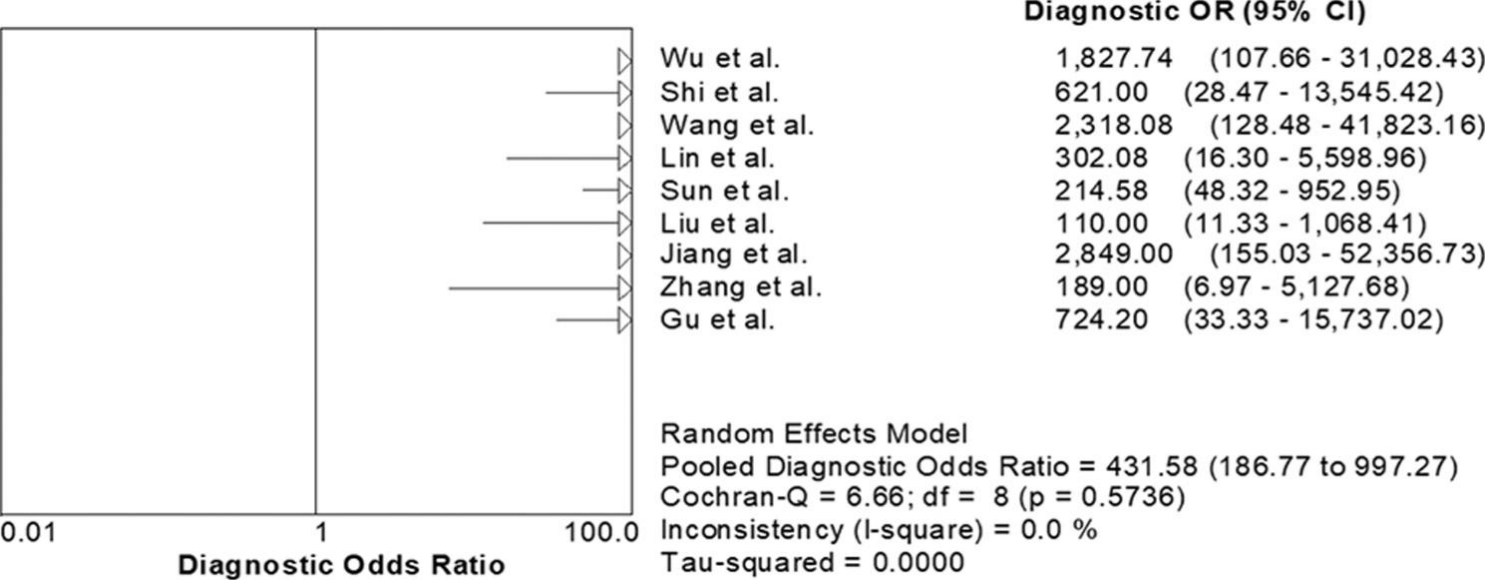
*I*^2^ test for the pooled diagnostic odds ratio (DOR). The value of *I*^2^ test for the pooled DOR indicated no heterogeneity between studies

**Fig. 5:**
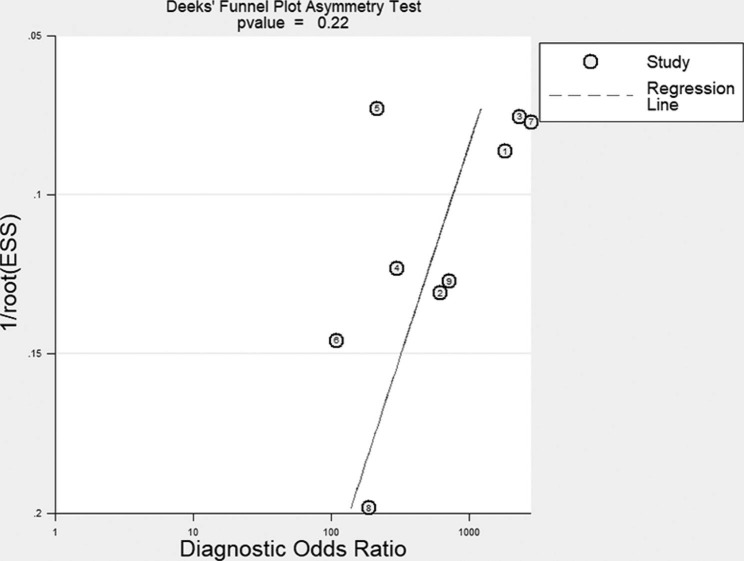
The Deek’s funnel plot for assessment of publication bias. No publication bias was found among the included studies

The value of *I*^2^ test for the pooled DOR was 0%, indicating no heterogeneity between studies (Fig. 4). The Deek funnel plot asymmetry test suggested no significant publication bias (*P* = 0.22) (Fig. 5).

### Subgroup analysis

**Fig. 6:**
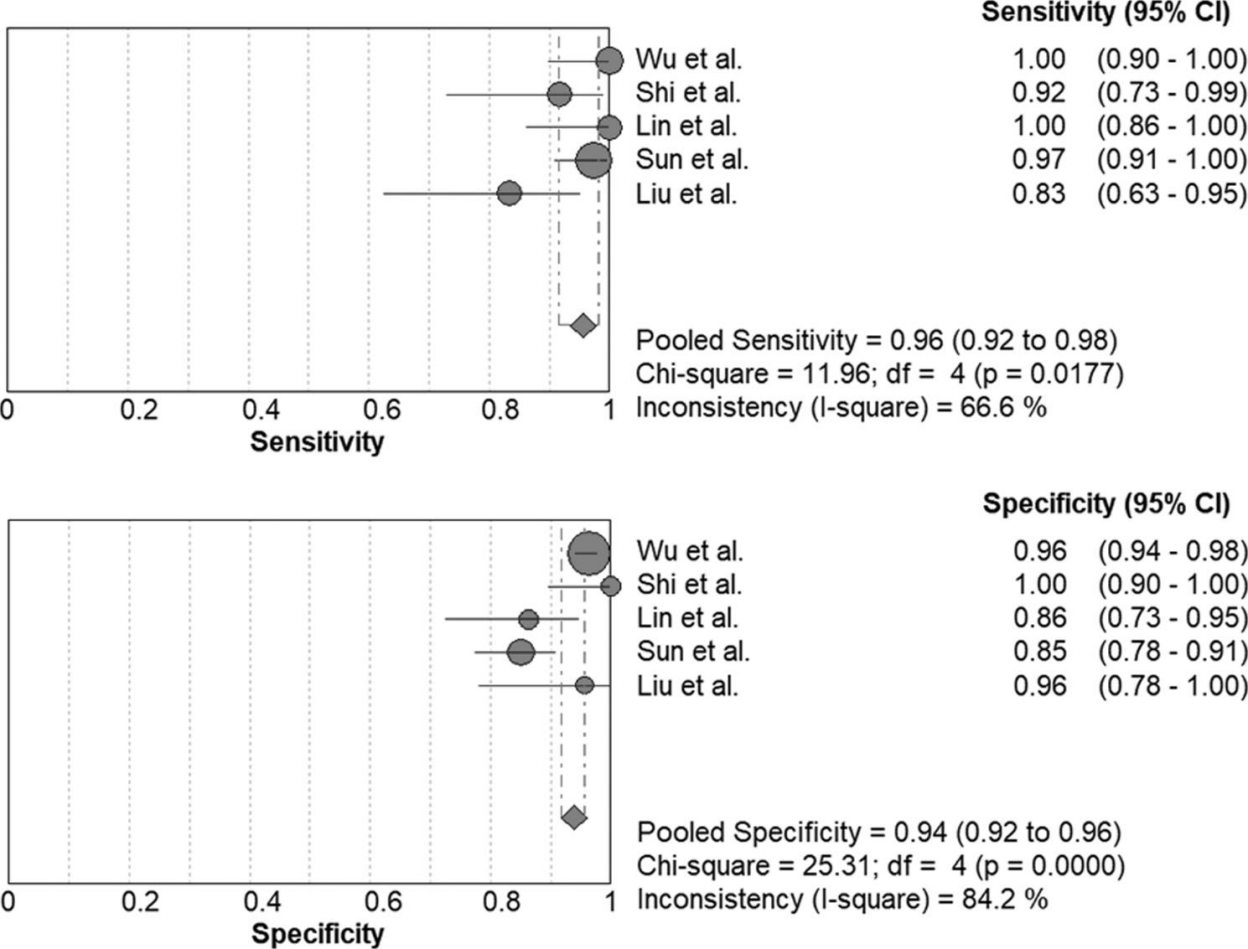
Forest plot of sensitivity and specificity for bronchoalveolar lavage fluid (BALF) metagenomic next-generation sequencing in diagnosis of Pneumocystis jirovecii pneumonia. The pooled sensitivity was 0.957 (95% CI, 0.917–0.981) and the pooled specificity was 0.939 (95% CI, 0.918–0.956)

As shown in Fig. 6, BALF mNGS had a pooled sensitivity of 0.957 (95% CI, 0.917–0.981) in the diagnosis of PJP. The pooled specificity, PLR, NLR, DOR, area under the SROC curve and Q* value were 0.939 (95% CI, 0.918–0.956), 13.043 (95% CI, 5.547–30.666), 0.061 (95% CI, 0.020–0.187), 287.50 (95% CI, 105.27–785.20), 0.9832 and 0.9435, respectively.

**Fig. 7:**
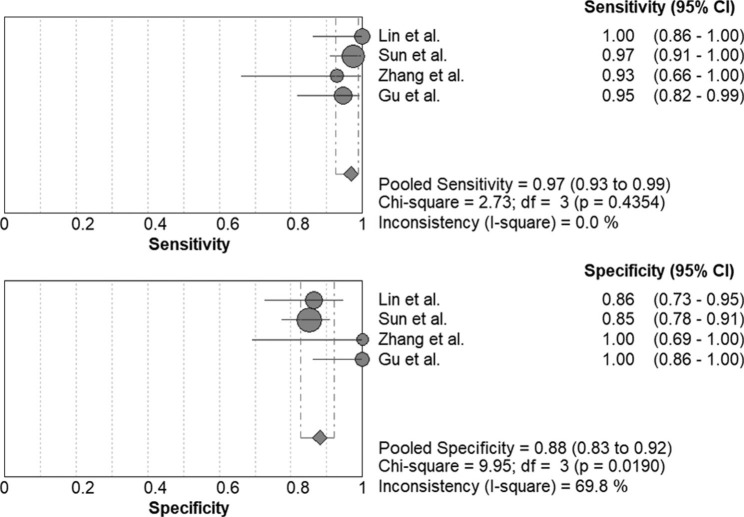
Forest plot of sensitivity and specificity for metagenomic next-generation sequencing in diagnosis of immunocompromised patients with Pneumocystis jirovecii pneumonia. The pooled sensitivity was 0.967 (95% CI, 0.925–0.989) and the pooled specificity 0.880 (95% CI, 0.827–0.922)

The area under the SROC curve of mNGS in the diagnosis of PJP in immunocompromised patients was 0.9852, and the Q* value was 0.9476. Overall sensitivity and specificity was 0.967 (95% CI, 0.925–0.989) and 0.880 (95% CI, 0.827–0.922), respectively (Fig. 7). The PLR was 7.169 (95% CI, 4.615–11.137), the NLR was 0.054 (95% CI, 0.026–0.115), and the DOR was 263.43 (95% CI, 83.940-826.73).

**Fig. 8:**
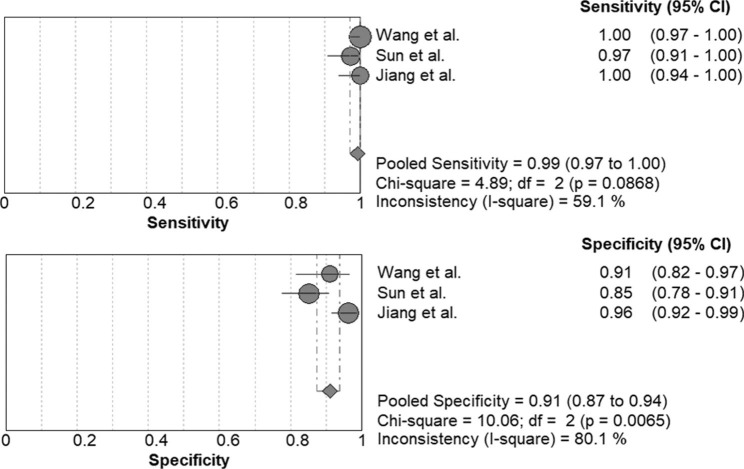
Forest plot of sensitivity and specificity for metagenomic next-generation sequencing in diagnosis of non-HIV patients with Pneumocystis jirovecii pneumonia. The pooled sensitivity was 0.992 (95% CI, 0.972–0.999) and the pooled specificity was 0.910 (95% CI, 0.873–0.939)

The accuracy of mNGS in the diagnosis of PJP in non-HIV patients was as follows: sensitivity 0.992 (95% CI, 0.972–0.999), specificity 0.910 (95% CI, 0.873–0.939), PLR 11.169 (95% CI, 5.170–24.130), NLR 0.018 (95% CI, 0.006–0.055), and DOR 772.97 (95% CI, 121.30-4925.9) (Fig. 8). The area under the SROC curve was 0.979, and the Q* value was 0.9353.

## Discussion

The clinical manifestations of PJP are not specific, and definite diagnosis requires direct tests of pathogens in lung tissues or lower respiratory secretions [1, 30]. Currently, diagnosis of PJP relies on the detection of pathogenic microorganisms by cytological staining, quantitative PCR, or immunofluorescence [31]. The mNGS is a novel assay that enables unbiased and detailed testing of the total RNA or DNA content of all known pathogens [15]. In the recent years, it has been increasingly used for pathogen diagnosis [32, 33]. To our knowledge so far there is no meta-analysis evaluating the clinical values of mNGS on PJP. In the present study, we enrolled 9 trials with a total of 1343 patients to assess the efficacy of mNGS for the diagnosis of PJP and conducted subgroup analyses to investigate the performance of mNGS in immunocompromised and non-HIV patients.

The current meta-analysis demonstrated that mNGS had a pooled sensitivity of 0.974 (95% CI 0.953–0.987) and specificity of 0.943 (95% CI 0.926–0.957) in diagnosis of PJP. The study by Liu et al. [22] showed a sensitivity of 0.833 for mNGS, however, only 24 cases of PJP were included. Of the 9 eligible studies, only the study by Zhang et al. [24] examined mNGS in sputum samples, but only 14 patients with PJP were enrolled. In the current meta-analysis, the included studies all had QUADAS scores greater than 10, suggesting that eligible trials were of high quality. The *I*^2^ test showed no heterogeneity between studies. The Deek funnel test suggested no potential publication bias.

The area under the SROC curve is used to estimate the overall performance of screening tests [34]. It summarises sensitivity and specificity, with values ranging 0.50–0.70 representing low diagnostic accuracy, 0.70–0.90 moderate accuracy, and > 0.90 high accuracy [35, 36]. In the present study, the area under the SROC curve was 0.987, and the Q* value was 0.951, indicating that mNGS has high accuracy for diagnosis of PJP. Our subgroup analysis showed that the overall efficacy of mNGS was similar in immunocompromised and non-HIV patients with PJP.

Positive results on BALF and lung biopsy specimens are considered as the “gold standard” for the diagnosis of PJP [12]. The main superiority of BALF is its proximity to the site of pulmonary infection, which is a good indication of the local lung environment [37, 38]. In the current meta-analysis, the combined sensitivity and specificity of BALF mNGS in the diagnosis of PJP were 0.957 and 0.939, respectively. The area under the SROC curve was 0.9832, and the Q* value was 0.9435. These results showed that BALF mNGS had excellent diagnostic value for PJP.

The genus Pneumocystis includes highly diverse fungal species that can cause serious pneumonia in patients with deficient immune systems. These fungi are strictly specific to the host species [39]. Five Pneumocystis species have been formally reported, including human-specific Pneumocystis jirovecii, rat-specific Pneumocystis carinii and Pneumocystis wakefieldiae, mouse-specific Pneumocystis murina, and rabbit-specific Pneumocystis oryctolagi [4]. Of the Pneumocystis genus, Pneumocystis jirovecii is the only species capable of infecting and reproducing in humans [40]. Pneumocystis jirovecii is an important fungal microorganism in immunocompromised patients [14]. PJP risk was usually related to individuals with HIV, bone marrow or solid organ transplant, malignancies including Hodgkin’s lymphoma and acute lymphoblastic leukemia, long-term usage of glucocorticoids, and severe malnutrition [41, 42]. Clinically significant PJP is found merely in hosts with acquired or congenital immunodeficiencies. PJP is not uncommon in immunocompromised cases, however, the pathogenesis is not fully understood [14]. In the present study, the pooled sensitivity of mNGS in the diagnosis of PJP in immunocompromised patients was 0.967, and the summary specificity was 0.880. The area under the SROC curve is 0.9852, and the Q* value is 0.9476, indicating that mNGS exhibited good diagnostic performance for PJP in immunocompromised hosts.

Delayed diagnosis of PJP was observed in non-HIV populations [3]. PJP in non-HIV and HIV patients differs in that there are more neutrophils with lower organism burden in non-HIV cases and fewer neutrophils with higher organism burden in HIV individuals [43]. Low organism burden in non-HIV patients attenuates the sensitivity of sputum staining [43, 44]. The clinical features of HIV complicated by PJP infection differ from those of immunodeficiency due to other causes. HIV patients often present with a longer course of PJP. Patients with immune dysfunction without HIV appear to have more severe manifestations and a higher risk of respiratory failure and death [42]. In the current meta-analysis, the pooled sensitivity of mNGS in the diagnosis of PJP in non-HIV patients was 0.992, and the summary specificity was 0.910. The area under the SROC curve is 0.979, and the Q* value is 0.9353. These data demonstrated that mNGS had favorable efficiency for non-HIV patients with PJP.

The present meta-analysis had several limitations. First, all included studies were not prospectively designed, which might potentially result in selection bias. Second, due to the limited number of eligible studies, different sequence platforms were used in our included trials. Third, some of the enrolled studies had small sample sizes, which may lead to insufficient capacity to assess diagnostic accuracy.

In summary, the current evidence indicates that mNGS has a good accuracy for the diagnosis of PJP. BALF mNGS exhibits excellent diagnostic performance for PJP. The mNGS is a promising tool for assessment of PJP in both immunocompromised and non-HIV patients.

## Data Availability

The datasets used and analysed during the current study available from the corresponding author on reasonable request.
